# *Herpes zoster*: A Review of Clinical Manifestations and Management

**DOI:** 10.3390/v14020192

**Published:** 2022-01-19

**Authors:** Anant Patil, Mohamad Goldust, Uwe Wollina

**Affiliations:** 1 Department of Pharmacology, Dr. DY Patil Medical College, Navi Mumbai 400706, India; anantd1patil@gmail.com; 2 Department of Dermatology, University Medical Center, Johannes Gutenberg University, 55131 Mainz, Germany; mgoldust@uni-mainz.de; 3 Department of Dermatology and Allergology, Städtisches Klinikum Dresden, 01067 Dresden, Germany

**Keywords:** *Varicella-zoster* virus, *Herpes zoster*, epidemiology, complications, treatment, prevention

## Abstract

The *Varicella-zoster* virus (VZV) or human herpes virus 3 is a neurotropic human alpha herpes virus responsible for chickenpox/varicella and shingles/*Herpes zoster* (HZ). This review will focus on HZ. Since HZ is secondary to varicella, its incidence increases with age. In children and youngsters, HZ is rare and associated to metabolic and neoplastic disorders. In adults, advanced age, distress, other infections (such as AIDS or COVID-19), and immunosuppression are the most common risk factors. HZ reactivation has recently been observed after COVID-19 vaccination. The disease shows different clinical stages of variable clinical manifestations. Some of the manifestations bear a higher risk of complications. Among the possible complications, postherpetic neuralgia, a chronic pain disease, is one of the most frequent. HZ vasculitis is associated with morbidity and mortality. Renal and gastrointestinal complications have been reported. The cornerstone of treatment is early intervention with acyclovir or brivudine. Second-line treatments are available. Pain management is essential. For (secondary) prophylaxis, currently two HZV vaccines are available for healthy older adults, a live attenuated VZV vaccine and a recombinant adjuvanted VZV glycoprotein E subunit vaccine. The latter allows vaccination also in severely immunosuppressed patients. This review focuses on manifestations of HZ and its management. Although several articles have been published on HZ, the literature continues to evolve, especially in regard to patients with comorbidities and immunocompromised patients. VZV reactivation has also emerged as an important point of discussion during the COVID-19 pandemic, especially after vaccination. The objective of this review is to discuss current updates related to clinical presentations, complications, and management of HZ.

## 1. Introduction

The *Varicella-zoster* virus (VZV) or human herpes virus 3 is the causative agent for both chickenpox/varicella and shingles/*Herpes zoster* (HZ). HZ represents a reactivation of VZV in the host and has gained interest because of variable clinical presentation, which is important in the differential diagnosis of diseases. Furthermore, HZ complications are potentially life-threatening. HZ reactivation has been reported as a possible adverse event after COVID-19 vaccination. Treatment options and prevention by vaccination are of clinical importance. HZ can present with different clinical manifestations, some with higher risk of complications. The literature related to HZ continues to evolve, especially in regard to patients with comorbidities and immunocompromised patients. VZV reactivation has emerged as an important point of discussion during the COVID-19 pandemic, especially after vaccination. With this background, in this review we discuss the current updates related to clinical presentations, complications, and management of HZ.

The literature was searched through PubMed and Google Scholar to retrieve relevant published articles on HZ. Clinical trials, clinical studies, review articles, systematic reviews, meta-analyses, case series, and case reports were considered for review. Keywords “*Varicella-zoster* virus”, “*Herpes zoster*”, “treatment AND *Herpes zoster*”, “prevention AND *Herpes zoster*” were used to search the articles. Articles published till 2021 along with reference lists of relevant articles were included for review.

## 2. Etiology

The VZV is a neurotropic human herpes virus belonging to the genus *alpha herpesviridiae*. It shows a worldwide distribution. The virus is responsible for primary infection resulting in varicella and HZ representing a reactivation of latent infection.

The VZV genome consists of about 125,000 base pairs of linear double-stranded DNA, and its nucleocapsid consists of 162 capsomers. The virus is highly cell-associated and only infects human cells, such as epithelial cells, T lymphocytes, and ganglionic neurons [1]. Virus entry into neural cells is mediated by heparan sulfate proteoglycan and the glycogen synthase kinase 3 (GSK-3) pathway [2]. Viral core glycoproteins B, H, and L participate in the core fusion complex. New virus particles can be released as soon as 9 to 12 h after cellular entry [3].

Varicella is acquired by airway contact with respiratory droplets or smears from vesicular varicella lesions and is one of the most contagious human disorders. The initial viral replication occurs in the respiratory tract, followed by invasion of local lymph nodes. Eventually, viremia occurs, associated with cutaneous vesicular eruptions. These lesions present a colorful picture of different stages, from early vesiculation to crusted lesions and possibly scars.

The incubation period for varicella varies between 10 and 21 days. Varicella is contagious from 1 to 4 days before the cutaneous rash and until all vesicular cutaneous lesions have dried up [4].

Varicella infection in pregnancy can spread via placenta, leading to fetal infection. Fetal varicella infection leads to disseminated life-threatening diseases. Vaccination protects the fetus [5].

While HZ is uncommon among children, the HZ risk could be diminished by 64% in children after vaccination for varicella, as shown in a Canadian study. Varicella vaccination in the youth does not seem to reduce HZ risk during aging [6].

After primary infection, the VZV virus becomes latent in nerval tissue. VZV has been detected in dorsal root ganglia, cranial nerve ganglia, and various autonomic ganglia in the enteric nervous system, and in astrocytes. Nectin-1, which is highly expressed in neurons, seems to be involved in viral entry of axons and cell bodies [7].

VZV-infected neurons overexpress anti-apoptotic proteins such as Bcl2 and Bcl-XL [8]. VZV latency has been associated to open reading frame (ORF) 63 [9]. VZV latency is controlled largely by cell-mediated immunity, and reactivation is considered a result of loss of such immune surveillance [10].

Upon reactivation, VZV replicates within cell bodies of neurons. In the next step, virus particles shed from the cell bodies down the nerve to the correlating dermatome. In the affected dermatome, the virus provokes inflammation and vesiculation. The pain caused by HZ is due to inflammation of nerves affected by VZV. HZ does not pose a risk to a developing fetus due to specific maternal antibodies that are transmitted diaplacental to the fetus [11].

## 3. Epidemiology

HZ occurs worldwide without seasonal variations of incidence. The incidence of HZ is age-dependent and ranges from 1.2 to 3.4 per 1000 persons per year among younger adults to 3.9–11.8 per 1000 persons per year in elderly patients (i.e., >65 years) [12]. According to a systematic review of studies from 2002–2018, the cumulative incidence has been estimated between 2.9–19.5 cases per 1000 population with female predominance [13].

Common risk factors for HZ are age > 50 years, immunosuppression, infections, and mental stress [14]. A meta-analysis of 16 studies till January 2021 confirmed that patients with diabetes mellitus also have a higher risk (pooled relative risk: 1.38; 95% confidence interval (CI): 1.21–1.57) [15].

A recently published (2021) Indian study reported a significant association between pediatric HZ and megaloblastic anemia. A major cause of megaloblastic anemia is vitamin B12 or folic acid deficiency [16].

According to the Global Burden of Disease database, the mortality rate due to HZ in patients >65 years ranges from 0.0022 to 82.21 per 100,000 population [17]. According to 2007 and 2008 HZ-outpatient incidence data from Germany, the annual mortality rate of HZ has been estimated as 0.29 (women) and 0.10 (males) per 100,000 patient years [18].

It is important to note possible heterogeneity in epidemiological data due to differences in reporting. It is possible that countries without efficient and effective reporting systems may not have lower numbers than those with efficient reporting systems.

## 4. Clinical Stages

Clinical symptoms appear in three stages—pre-eruptive, acute exsudative, and chronic [12]. The pre-eruptive stage presents with burning or pain within the affected dermatome at least 2 days prior to cutaneous eruptions. Noncutaneous symptoms such as experiencing headaches, general malaise, and photophobia may also be present.

In the acute eruptive phase, multiple umbilicated and painful vesicles develop. The vesicles often burst, ulcerate, and eventually dry out. This is the most contagious stage. Pain is often severe and unresponsive to nonsteroidal pain medications. The acute eruptive phase may last 2–4 weeks. Pain can continue longer.

Chronic HZ infection is characterized by severe pain that lasts >4 weeks. Patients experience dysesthesias, paresthesias, and sometimes shock-like sensations. The pain is disabling and may last for several months.

In most patients, diagnosis is made clinically. Due to variable clinical presentation and atypical cases, the diagnosis of HZ may be challenging in some patients [19].

Polymerase chain reaction (PCR) is useful for confirmation of suspected HZ-type pain without a rash.

## 5. Special Clinical Patterns

### 5.1. HZ Ophthalmicus (HZO)

HZO is defined as VZV involvement of the ophthalmic division (V1) of the trigeminal nerve (V). About 50–85% of HZO cases experience ocular complaints such as conjunctivitis, uveitis, episcleritis, keratitis, or retinitis [20,21]. Rare manifestations are the sterile iris abscess [22], oculomotoric nerve palsy [23], superior orbital fissure syndrome [24], orbital apex syndrome [25], and isolated nonreactive mydriasis [26].

HZO is an ophthalmologic emergency. There is a risk of vision loss. The main risk factors for HZO include age >50 years and immunosuppression [24,27,28]. On multivariate analysis, severe vision loss has been associated with advanced age (hazard ratio (HR): 1.1), immunosuppression by drugs or disease (HR: 3.1), poor presenting visual acuity (HR: 2.8), and uveitis (HR: 4.8) [21].

Feared possible complications of HZO include the following:

- Orbital phlegmon with a risk of secondary blindness;
- Acute retinal necrosis with possible visual loss;
- Anterior uveitis;
- Epithelial punctate keratitis;
- Corneal inflammation and opacification with visual impairment [21].

Patients with HZO have a 1.3- to 4-fold increased risk of cerebrovascular events following the disease—even in younger patients [29].

### 5.2. Ramsay Hunt Syndrome

Ramsay Hunt syndrome is a less common subtype of HZ involving geniculate ganglion and facial nerve (7th cranial nerve) representing <1% of all HZ cases. Typical symptoms are unilateral facial palsy, otalgia, and painful vesicles on the auricle and/or external auditory canal. Postherpetic neuralgia (PHN), however, is uncommon. Concomitant involvement of the vestibulocochlear (8th cranial nerve) or trigeminal nerve (5th cranial nerve) has commonly been observed [30]. This may translate into dizziness, tinnitus, hearing impairment, or facial pain. Glossopharyngeal (9th cranial nerve) or vagal (10th cranial nerve) involvement may lead to symptoms like dysphagia, hoarseness, or even cardiac manifestation [31,32].

### 5.3. Disseminated HZ (HZ Generalisatus)

Disseminated HZ is uncommon. It is seen more often in immunocompromised patients. Rarely, a co-infection with HZ and herpes simplex might be responsible [33].

### 5.4. Deep HZ

The infection of stromal cells is well known from HZO but underreported from other sites. The most common non-ocular areas involved are the perianal region and adjacent buttocks, where ulcerations frequently occur. However, internal organs such as the gastrointestinal system may also be involved [34]. The major risk factor for deep HZ is immunosuppression [35].

### 5.5. Purpuric HZ

Purpuric HZ is rare. The major differential diagnosis is cutaneous vasculitis. A diagnostic biopsy is helpful in such cases [36].

### 5.6. Central Nervous System HZ

HZ may affect the central nervous system (CNS). Immunocompromised patients, such as AIDS/ HIV patients, are prone for CNS manifestation. By vessel wall magnetic resonance (MR) imaging, arterial vasculitis intra- and extracranial blood vessels can be detected. Typical findings are vessel wall enhancement, vessel wall thickening, edema, and perivascular enhancement [37]. In a nationwide study from Denmark, the incidence of CNS HZ was estimated at 5.3/1,000,000 per year. The median age was 75 years, with frequent association to immunocompromising conditions. CNS HZ can cause personality changes, confusion, headache, nausea, and gait problems [38].

VZV vasculopathy can lead to cerebral infarcts and acute stroke [39,40,41]. VZV-induced thoracic myelomyelitis can cause paraplegia [42].

## 6. Complications

### 6.1. Postherpetic Neuralgia

Postherpetic neuralgia (PHN) is a frequent complication of HZ characterized by intense pain. While the usual HZ pain lasts 2 to 4 weeks, PHN is defined as pain persistent after 4 or 9 weeks. How VZV causes acute pain and the mechanisms underlying the transition to PHN are far from clear. Mechanical allodynia and thermal hyperalgesia are part of PHN [43].

Several neuroimaging studies detected abnormalities in brain structure and aberrant brain activities in sensory- and emotional-linked brain districts. Changes in gray matter volume and intrinsic functional connectivity in certain areas are closely associated with chronification from HZ to PHN [44].

PHN has been characterized by abnormalities within default mode network (DMN), DMN–salience network (SN), and SN–basal ganglia network connectivity relative to healthy controls. Available data argue for an acquired network-level imbalance in PHN [45].

Medication-resistant PHN is a further challenge. A Chinese study evaluated potential risk factors and identified subacute HZ (vs. acute HZ; odds ratio (OR): 9.0), severe lesions (vs. mild lesions; OR: 3.8), and depressed mood (OR: 1.1) as risk factors [46].

Computed tomography (CT)-guided radiofrequency ablation of the cervical dorsal root ganglia decreased visual analogue scale (VAS) pain scores and cutaneous sensations. Allodynia was significantly diminished [47].

### 6.2. Meningitis Retention Syndrome

Meningitis retention syndrome is a rare condition. It is defined as acute urinary retention due to aseptic meningitis—a mild form of acute disseminated encephalomyelopathy. That would explain why these patients also suffer from fever, headache, stiff neck, and minor pyramidal signs. The bladder is initially areflexic [48,49].

### 6.3. Acute Colonic Pseudo-Obstruction

Acute colonic pseudo-obstruction (Ogilvie syndrome) is a rare complication of HZ. Only 28 patients have been published between 1950 and 2008. There was a clear male predominance (76%). The mean age was 61 years [50].

### 6.4. Keloids and Other Types of Isotopic Response

Keloids are benign connective tissue tumors developing due to an aberration during wound-healing. Keloids have occasionally been observed on healing HZ lesions, especially in ethnic skin, as an isotopic skin reaction [51,52].

Specific infiltrates of chronic B-cell lymphocytic leukemia have developed in formerly HZ-affected areas [53,54]. These are examples of immunocompromised districts of skin that are at risk for the development of secondary dermatoses [55,56].

### 6.5. Pseudohernia Formation and Cysts

Unilateral abdominal bulging is a kind of pseudohernia formation. It may develop due to paresis of abdominal muscles. The combination of unilateral abdominal bulging and herpetic vesicles indicates abdominal pseudohernia due to HZ. In some cases, the rash may develop after the bulging [57].

Verma et al. described postzosteric pseudoherniation of the skin in affected limbs. Histological examination surprisingly revealed neo-lymphangiogenesis to be responsible [58]. Rahmatpour Rokni et al. reported the formation of cutaneous cysts after HZ with PHN [59].

### 6.6. Erythema Multiforme

Erythema multiforme (EM) represents a hypersensitivity reaction to infections or drugs. It is characterized by polymorphous skin eruptions such as macules, papules, and characteristic “targetoid” lesions. Eight patients with HZ-associated EM have been described so far [60,61].

### 6.7. Vasculitis

VZV can cause a vasculitis or vasculopathy of different types. CNS arteritis has been mentioned above. Retinal vasculitis is a severe, but rare complication of HZO treated by antiviral medication [62].

Biopsies of giant cell arteritis demonstrated VZV in affected vessels in about two-thirds of patients. HZ causes vasculopathy most commonly in the arterial adventitia, followed by media and intima. Occasionally, VZV has been detected in granulomatous aortitis and temporal arteritis [63]. A retrospective investigation on several thousand patients using Medicare and Truven Health Analytics MarketScan suggested an increased risk of giant cell arteritis in HZ patients [64]. In contrast, a geo-epidemiological study did not confirm an association between VZV and giant cell arteritis [65].

Leukocytoclastic vasculitis has been described in the same dermatome as that affected by HZ [66,67], while unilateral small-vessel vasculitis has occasionally been observed in HZ with and without sarcoidosis [68,69].

A very rare complication of HZ is IgA vasculitis with gastrointestinal symptoms [70].

### 6.8. Recurrent HZ

Recurrent HZ is a possible complication in elderly and immunocompromised patients. In patients ≥45 years of age, a recurrence rate of 3.9% has been estimated [71].

Patients ≥45 years of age with rheumatic disorders and who are taking disease-modifying drugs (DMARDS) are another group at risk. In a large trial with 254,065 eligible participants, risk of HZ was higher in those who used cyclophosphamide (adjusted hazard ratio (aHR): 2.7; 95% CI: 1.89, 3.83), azathioprine (aHR: 1.6; 95% CI: 1.07, 2.30), and hydroxychloroquine (aHR: 1.4; 95% CI: 1.11, 1.83) but was not elevated by methotrexate, sulfasalazine, or leflunomide [72].

Recurrent HZ has been observed in chronic urticaria patients treated with cyclosporin-A after COVID-19 vaccination [73].

### 6.9. HZ and Occult Neoplasia

In a systematic review and meta-analysis of published studies, the pooled relative risk for any cancer in HZ patients was 1.42 (95% CI: 1.18, 1.71). It raised to 1.83 (95% CI: 1.17, 2.87) at one year after HZ with the highest estimates for hematological malignancies [74]. This has been substantiated by another case control study from the UK [75]. In a population-based study from Taiwan, the risk of subsequent cancer was elevated within two years after HZ. The most common cancer to be observed was lung cancer in this setting [76]. An especially severe or atypical HZ course may be a sign of underlying neoplasia [77].

According to a prospective cohort, patients with both hematological and solid cancers have increased risk of zoster as compared to those without cancer. Risk of HZ increased even before the diagnosis of hematological cancer (but not before diagnosis of solid cancers). Moreover, as compared to people without cancer, patients with solid cancer receiving chemotherapy had higher risk of HZ than did those not receiving it [78]. These observations need to be considered in the management of HZ in patients with cancer.

### 6.10. VZV Reactivation and COVID-19

Varicella-like disease and HZ may be cutaneous symptoms in patients affected by COVID-19 [79,80,81].

A potential adverse event after COVID-19 vaccination is HZ. All available vaccines have been found to potentially reactivate VZV. In a nationwide analysis with tozinameran (BNT162b2 mRNA vaccine (BioNTech, Mainz, Germany; Pfizer, New York, NY, USA)) from Israel, HZ infection had a risk ratio of 1.43 (15.8 events per 100,000 persons) [82].

In the European EudraVigilance database, over 4100 cases of HZ are reported following the tozinameran/Comirnaty (BioNTec-Pfizer, New York, NY, USA) vaccine, accounting for 1.3% of total reported events following this vaccination. For spikevax (mRNA-1273; Moderna, Cambridge, MA, USA), 590 (0.7%) cases have been reported, for Covishield/Vaxzevria (CHADOX1 NCOV-19; Oxford-AstraZeneca, Cambridge, UK), 2143 (0.6%) cases, and for COVID-19 vaccine (AD26.COV2.S; Janssen, Beerse, Belgium), 59 cases (0.3%) [83]. A total of 2512 HZ cases (1.3% of total reported events) after tozinameran/corminaty, 1763 (0.9%) after spikevax, and 302 (0.7%) after COVID-19 vaccine are reported in the United States Vaccine Adverse Event Report System (VAERS) [84].

A systematic review described 91 patients with HZ following COVID-19 vaccination. Important comorbidities in these patients included hypertension, autoimmune disorders, and immunosuppression [85]. In a recent narrative review on HZ after COVID-19 vaccination by Iwanaga, et al., 399 cases are reported. The affected dermatomes in these patients did not differ from the regular distribution of HZ. Some patients with a history of VZV vaccination developed HZ after COVID-19 vaccination [86].

## 7. Treatment with Biologicals

In a retrospective cohort study with >2000 patients suffering from ankylosing spondylitis, treatment with TNF-alpha inhibitor infliximab did not increase the risk for HZ [87].

In patients with psoriasis, treatment with infliximab and etanercept increased the risk for HZ with an OR of 2.43 and 1.65, respectively [88].

JAK inhibitors bear an increased risk for HZ reactivation. Upadacitinib is an oral Janus kinase (JAK) inhibitor approved for rheumatoid arthritis. The incidence rate of HZ was 3.0 (2.6 to 3.5) for 15 mg upadacitinib and 5.3 (4.5 to 6.2) for the doubled dosage [89].

## 8. Medical Treatment

The standard therapy of HZ is acyclovir (ACV) and its prodrug valacyclovir or brivudine (Table 1). Oral valacyclovir offers the advantage of a three- to fivefold increase in acyclovir bioavailability [90]. ACV and valacyclovir are processed to nucleoside analogues, which specifically block viral DNA replication in affected cells. Mutations in the viral thymidine kinase and/or DNA polymerase are responsible for ACV resistance [91].

A rare adverse event of ACV and other antiviral medications therapy is renal toxicity. In patients with renal impairment a dose reduction may be necessary. Only brivudin has no renal toxicity. An absolute contraindication for brivudine, however, is any treatment with 5-fluorouracil or other 5-fluoropyrimidine compounds within the last 4 weeks [43].

In case of ACV-resistant HZ, famciclovir is an alternative. Valganciclovir is a valine ester prodrug of gancyclovir that is orally administrable, and it has recently shown activity against VZV [92].

Helicase-primase inhibitors (HPIs) such as amenamevir inhibit the progression of the replication fork, an initial step in DNA synthesis to separate the double strand into two single strands. Amenamevir is the first compound of this new class and has received approval for HZ treatment in Japan [93].

Prevention of VZV reactivation is of particular importance in patients with immunosuppression. In a retrospective trial among 45 US transplant centers, 2 × 400 mg ACV/d was the most common dose used for HZ prevention, but low-dose famcilovir seems to be effective as well [94].

Ethanol extract of *Elaeocarpus sylvestris var. ellipticus* ES has been reported to inhibit the expression of VZV replication-related genes and cell death due to viral infection in vitro. It also reduces peripheral and central inflammatory pain [95]. ES16001 is a tablet consisting of 50% ethanol extract of ES (Genencell Co. Ltd., Yongin, Gyeonggi-do, Korea), which seems to have a potential for protection against reactivation of VZV virus, but further investigations are needed [96].

## 9. Vaccination

HZ vaccines aim to prevent activation of HZ and the development of PHN. Currently, two HZ vaccines are available for healthy older adults, a live attenuated VZV vaccine (Zostavax; Merck, Kenilworth, NJ, USA) and a recombinant adjuvanted VZV glycoprotein E subunit vaccine (Shingrix, GlaxoSmithKline, London, UK). Live attenuated vaccine had been the standard vaccine for years. The safety and efficacy of both vaccines has been demonstrated in clinical trials in immunocompetent healthy adults, in selected immunocompromised patients, and in patients with immune disorders. Recombinant HZV vaccine is more effective for prevention of HZ compared to live attenuated HZV vaccine. Recombinant HZV vaccine is nonreplicating and is therefore safe also for immunocompromised persons [97,98].

A randomized controlled trial with 617 patients on tumor necrosis factor-alpha inhibitors (NCT02538341) demonstrated a favorable safety of the live attenuated vaccine in this particular patient’s group [99].

The NCT02581410 trial investigated the effect of recombinant HZV (Shingrix, GSK) in elderly patients who either had been vaccinated with live attenuated HZV vaccine ≥5 years before or were HZV naive. Recombinant HZV induced a strong humoral and cell-mediated immune response that persisted above prevaccination levels for 12 months following the second dose, irrespective of previous vaccination [100].

A Chinese study analyzed the potential public health impact of recombinant HZV vaccination, compared with the status quo of no vaccination, in individuals ≥50 years of age in Beijing. Mass vaccination with recombinant HZV was estimated to prevent >430,000 HZ cases and >51,000 PHN cases compared with no vaccination. The authors suggested that >14,000 hospitalizations and >1,000,000 outpatient visits could be avoided. Patients between 50 and 59 years had the greatest overall reduction in HZ cases, complications, and related healthcare resource use [101].

A claims-based control trial from the US estimated the effectiveness of recombinant HZ vaccine in patients between 50 and 79 years old. The study estimated the incidence rate of HZ as 258.8 cases per 100,000 person-years in vaccinated persons, compared with 893.1 in unvaccinated controls. The recombinant HZV vaccine effectiveness reached 85.5% [102].

Another large cohort study from the US in patients ≥65 years estimated a recombinant vaccine effectiveness of 70.1% and 56.9% for two and one doses, respectively. Two-dose vaccine effectiveness against PHZ reached 76.0% [103].

A rare adverse event after recombinant HZV vaccination is myelin oligodendrocyte glycoprotein-related optic neuritis [104].

## 10. Prevention and Treatment of Postherpetic Neuralgia (PHN) (except Vaccination)

While nonsteroidal anti-inflammatory drugs (NSAID) are not effective in PHN [105], tricyclic antidepressants, selective serotonin reuptake inhibitors, gabapentin, or pregabalin represent the treatment of first choice. Topical capsaicin ointment or transdermal drug delivery systems with lidocain may be helpful. A physical alternative is transcutaneous neural electrostimulation (TENS) [106].

Under investigation are donepezil, ambroxol, statins, and peroxisome proliferator-activated receptor (PPAR) agonists (ATx086001), topical cannabinoid receptor agonist N-palmitoyl ethanolamin, local injections with botulinum toxin, and invasive neurostimulatory approaches [107,108].

A systematic review analyzed strategies for the prevention of PHN after HZ disease. The incidence of PHN was lower with continuous epidural block with local anesthetics and steroids than with antiviral agents with subcutaneous injection of local anesthetics and steroids or antiviral agents with intracutaneous injection of local anesthetics and steroids at 3 months after acute HZ disease [109].

## 11. Conclusions

HZ represents a reactivation of VZV in the host. Patients with HZ infection can present with varied manifestations and several complications. Special clinical presentations include HZ opthalmicus, Ramsay Hunt syndrome, disseminated HZ, deep HZ, purpuric HZ, and central nervous system HZ. It can lead to postherpetic neuralgia or other complications. Recurrent HZ is possible in elderly and immunocompromised patients. Patients with hematological and solid cancers have increased risk of HZ. Patients with solid cancer receiving chemotherapy have higher risk of HZ than do those not receiving it. The standard therapy of HZ is acyclovir (ACV) and its prodrug valacyclovir or brivudine. HZ vaccines aim to prevent activation of HZ and the development of PHN. Currently, two HZ vaccines are available for healthy older adults.

## Figures and Tables

**Table 1:** Medical treatment of HZ.

Drug	Dosage	Remarks
Acyclovir	Adults: 5 × 800 mg/day p.o.	Limited bioavailability
	3 × 500 mg/day i.v.	In uncomplicated HZ
	3–5 × 10 mg/kg/day	In severe HZ, in case of immunosuppression
		for 10 day, usually 5–7 days
	Children: 3 × 10 mg/kg/day	Maximum daily dosage 2,5 g
Brivudin	Adults: 125 mg once a day p.o.	For 5 days.
Valaciclovir	Adults: 3 × 1000 mg/day p.o.	For 7 days
Famciclovir	Adults: 3 × 250–500 mg/day	2nd line in ACV-resistant patients

## Data Availability

Data sharing not applicable.
